# Supplementary material for: Development of a Novel In Silico Classification Model to Assess Reactive Metabolite Formation in the Cysteine Trapping Assay and Investigation of Important Substructures
Source: Biomolecules. 2024 Apr 30;14(5):535. doi: 10.3390/biom14050535 (PMC11117661; doi:10.3390/biom14050535)
Supplement: Supplementary file 1 [file biomolecules-14-00535-s001.zip › biomolecules-2958830-supplementary.pdf]

Supporting Information

Development of a novel *in silico* classification model to assess reactive metabolite formation in the cysteine trapping assay and investigation of important substructures

Yuki Umemori[1], Koichi Handa[1]*, Saki Yoshimura[1], Michiharu Kageyama[1], Takeshi Iijima[1]

[1]DMPK Research Department, Teijin Institute for Bio-medical Research, TEIJIN PHARMA LIMITED, 4-3-2 Asahigaoka, Hino-shi, Tokyo 191-8512, Japan

*** Correspondence:**
Dr. Koichi Handa
koichi.handa@axcelead-twp.com

Table S1. Dataset for cysteine trapping assay (475 compounds).

Compound Name	RI integrated area (count)	Positive (P) / Negative (N)
Acetaminophen	1791.7	P
Aminopyrine	44.3	N
Amlodipine	42.9	N
Amodiaquine	346.7	N
Atorvastatin	4464.4	P
Benzbromarone	4516.4	P
Caffeine	22.4	N
Carbamazepine	61.8	N
Clopidogrel	708.2	N
Clozapine	1286.4	P
Diclofenac	1230.1	P
Donepezil	38.4	N
Erythromycin	10.3	N
Fluoxetine	153.8	N
Flutamide	680.4	N
Furosemide	768.1	N
Ibrutinib	1603.8	P
Imipramine	2689.4	P
Indomethacin	89.0	N
Levofloxacin	150.3	N
Olanzapine	4161.2	P
Osimertinib	2319.2	P
Phenytoin	34.7	N
Pioglitazone	105.8	N
Pravastatin	25.4	N
Propranolol	3667.2	P
Ritonavir	593.9	N
Rosiglitazone	1593.7	P
Sulfamethoxazole	89.0	N

Compound Name	RI integrated area (count)	Positive (P) / Negative (N)
Tacrine	243.5	N
Tamoxifen	162.1	N
Ticlopidine	646.0	N
Tienilic acid	3783.7	P
Troglitazone	521.7	N
Troleandomycin	68.8	N
Verapamil	1087.2	P
Warfarin	79.6	N
Zafirlukast	263.4	N
Zomepirac	217.5	N
In-house 1	51962.0	P
In-house 2	868.6	N
In-house 3	128939.9	P
In-house 4	734.6	N
In-house 5	153.0	N
In-house 6	41741.7	P
In-house 7	219.6	N
In-house 8	713.3	N
In-house 9	569.0	N
In-house 10	836.3	N
In-house 11	1483.2	P
In-house 12	227.2	N
In-house 13	120.3	N
In-house 14	18536.6	P
In-house 15	8275.8	P
In-house 16	465.5	N
In-house 17	277.3	N
In-house 18	7079.9	P
In-house 19	550.8	N
In-house 20	2336.0	P

Compound Name	RI integrated area (count)	Positive (P) / Negative (N)
In-house 21	1016.9	P
In-house 22	252.1	N
In-house 23	756.9	N
In-house 24	5760.5	P
In-house 25	387.7	N
In-house 26	1683.4	P
In-house 27	201.7	N
In-house 28	394.4	N
In-house 29	147.7	N
In-house 30	1084.6	P
In-house 31	471.4	N
In-house 32	286.3	N
In-house 33	436.0	N
In-house 34	635.4	N
In-house 35	112.7	N
In-house 36	2405.2	P
In-house 37	471.6	N
In-house 38	435.8	N
In-house 39	526.1	N
In-house 40	400.7	N
In-house 41	2561.3	P
In-house 42	466.8	N
In-house 43	333.2	N
In-house 44	315.7	N
In-house 45	263.7	N
In-house 46	332.6	N
In-house 47	391.9	N
In-house 48	260.9	N
In-house 49	646.6	N
In-house 50	423.6	N

Compound Name	RI integrated area (count)	Positive (P) / Negative (N)
In-house 51	918.3	N
In-house 52	686.0	N
In-house 53	8498.4	P
In-house 54	1516.7	P
In-house 55	1438.0	P
In-house 56	371.6	N
In-house 57	31509.3	P
In-house 58	376.3	N
In-house 59	442.8	N
In-house 60	440.2	N
In-house 61	721.9	N
In-house 62	262.7	N
In-house 63	205.0	N
In-house 64	291.3	N
In-house 65	451.0	N
In-house 66	361.5	N
In-house 67	205.5	N
In-house 68	248.3	N
In-house 69	362.4	N
In-house 70	6152.5	P
In-house 71	114.5	N
In-house 72	351.9	N
In-house 73	533.4	N
In-house 74	370.1	N
In-house 75	101.1	N
In-house 76	1517.4	P
In-house 77	2246.7	P
In-house 78	1436.3	P
In-house 79	210.7	N
In-house 80	25.3	N

Compound Name	RI integrated area (count)	Positive (P) / Negative (N)
In-house 81	83.2	N
In-house 82	844.1	N
In-house 83	133.7	N
In-house 84	96.1	N
In-house 85	203.5	N
In-house 86	176.3	N
In-house 87	107.3	N
In-house 88	572.3	N
In-house 89	91.7	N
In-house 90	36487.1	P
In-house 91	180.8	N
In-house 92	466.4	N
In-house 93	1362.1	P
In-house 94	1138.2	P
In-house 95	377.9	N
In-house 96	206.7	N
In-house 97	2788.4	P
In-house 98	538.0	N
In-house 99	53.1	N
In-house 100	5858.3	P
In-house 101	270.1	N
In-house 102	486.9	N
In-house 103	414.8	N
In-house 104	215.8	N
In-house 105	221.9	N
In-house 106	339.4	N
In-house 107	805.1	N
In-house 108	763.7	N
In-house 109	6817.1	P
In-house 110	1155.6	P

Compound Name	RI integrated area (count)	Positive (P) / Negative (N)
In-house 111	2047.0	P
In-house 112	633.2	N
In-house 113	6784.0	P
In-house 114	2306.2	P
In-house 115	107.1	N
In-house 116	3306.0	P
In-house 117	1370.6	P
In-house 118	142.6	N
In-house 119	132.7	N
In-house 120	22.0	N
In-house 121	2287.6	P
In-house 122	5489.1	P
In-house 123	10185.7	P
In-house 124	939.7	N
In-house 125	1435.3	P
In-house 126	840.9	N
In-house 127	1769.2	P
In-house 128	646.3	N
In-house 129	10937.1	P
In-house 130	9382.6	P
In-house 131	1716.6	P
In-house 132	957.1	N
In-house 133	2082.3	P
In-house 134	705.8	N
In-house 135	1673.1	P
In-house 136	217.9	N
In-house 137	7733.3	P
In-house 138	1057.4	P
In-house 139	2770.4	P
In-house 140	1421.9	P

Compound Name	RI integrated area (count)	Positive (P) / Negative (N)
In-house 141	2479.0	P
In-house 142	1706.6	P
In-house 143	4185.0	P
In-house 144	1379.7	P
In-house 145	4424.8	P
In-house 146	20.3	N
In-house 147	3571.5	P
In-house 148	1016.3	P
In-house 149	2120.7	P
In-house 150	1663.9	P
In-house 151	985.7	N
In-house 152	6627.2	P
In-house 153	1046.8	P
In-house 154	280.4	N
In-house 155	4.0	N
In-house 156	599.7	N
In-house 157	3604.6	P
In-house 158	417.3	N
In-house 159	444.2	N
In-house 160	639.3	N
In-house 161	73.0	N
In-house 162	1675.7	P
In-house 163	17.4	N
In-house 164	2.7	N
In-house 165	1168.7	P
In-house 166	5376.6	P
In-house 167	183.2	N
In-house 168	1265.0	P
In-house 169	549.7	N
In-house 170	647.1	N

Compound Name	RI integrated area (count)	Positive (P) / Negative (N)
In-house 171	85.7	N
In-house 172	4966.4	P
In-house 173	881.7	N
In-house 174	4055.7	P
In-house 175	8226.7	P
In-house 176	652.8	N
In-house 177	50.2	N
In-house 178	512.5	N
In-house 179	519.6	N
In-house 180	2802.7	P
In-house 181	846.5	N
In-house 182	1342.1	P
In-house 183	3817.2	P
In-house 184	196.6	N
In-house 185	1596.1	P
In-house 186	110.7	N
In-house 187	78.6	N
In-house 188	931.6	N
In-house 189	1635.7	P
In-house 190	4325.7	P
In-house 191	6513.4	P
In-house 192	1259.3	P
In-house 193	3623.5	P
In-house 194	2021.4	P
In-house 195	728.7	N
In-house 196	28779.4	P
In-house 197	29.6	N
In-house 198	44.3	N
In-house 199	317.3	N
In-house 200	15.4	N

Compound Name	RI integrated area (count)	Positive (P) / Negative (N)
In-house 201	2939.1	P
In-house 202	24.1	N
In-house 203	3130.7	P
In-house 204	457.0	N
In-house 205	4338.6	P
In-house 206	178.7	N
In-house 207	1865.7	P
In-house 208	1345.6	P
In-house 209	744.4	N
In-house 210	416.8	N
In-house 211	1419.0	P
In-house 212	5395.1	P
In-house 213	1949.8	P
In-house 214	686.9	N
In-house 215	336.3	N
In-house 216	717.6	N
In-house 217	9270.8	P
In-house 218	6421.7	P
In-house 219	535.5	N
In-house 220	618.0	N
In-house 221	373.5	N
In-house 222	1945.6	P
In-house 223	2225.9	P
In-house 224	6416.8	P
In-house 225	319.3	N
In-house 226	6315.0	P
In-house 227	1450.7	P
In-house 228	6915.6	P
In-house 229	58.9	N
In-house 230	9632.8	P

Compound Name	RI integrated area (count)	Positive (P) / Negative (N)
In-house 231	3757.8	P
In-house 232	32806.5	P
In-house 233	381.6	N
In-house 234	3729.8	P
In-house 235	5257.2	P
In-house 236	668.7	N
In-house 237	3053.2	P
In-house 238	5852.9	P
In-house 239	350.1	N
In-house 240	456.0	N
In-house 241	538.4	N
In-house 242	669.9	N
In-house 243	5438.2	P
In-house 244	7636.5	P
In-house 245	24082.9	P
In-house 246	3637.6	P
In-house 247	522.5	N
In-house 248	47.0	N
In-house 249	49.7	N
In-house 250	44.7	N
In-house 251	140.5	N
In-house 252	165.3	N
In-house 253	324.7	N
In-house 254	104.0	N
In-house 255	263.9	N
In-house 256	4002.9	P
In-house 257	7790.0	P
In-house 258	512.7	N
In-house 259	3378.3	P
In-house 260	72.0	N

Compound Name	RI integrated area (count)	Positive (P) / Negative (N)
In-house 261	40.2	N
In-house 262	958.5	N
In-house 263	4807.9	P
In-house 264	298.0	N
In-house 265	3179.1	P
In-house 266	2672.4	P
In-house 267	6296.2	P
In-house 268	481.9	N
In-house 269	6831.8	P
In-house 270	4285.5	P
In-house 271	105.0	N
In-house 272	24248.2	P
In-house 273	60837.2	P
In-house 274	556.5	N
In-house 275	2505.7	P
In-house 276	29282.3	P
In-house 277	393.7	N
In-house 278	394.5	N
In-house 279	4490.1	P
In-house 280	1222.8	P
In-house 281	70.1	N
In-house 282	4195.0	P
In-house 283	1722.3	P
In-house 284	2266.7	P
In-house 285	670.8	N
In-house 286	847.8	N
In-house 287	278.8	N
In-house 288	6777.8	P
In-house 289	1183.0	P
In-house 290	4267.1	P

Compound Name	RI integrated area (count)	Positive (P) / Negative (N)
In-house 291	115.9	N
In-house 292	4672.3	P
In-house 293	20944.8	P
In-house 294	25974.3	P
In-house 295	7221.3	P
In-house 296	215.7	N
In-house 297	2196.1	P
In-house 298	3796.6	P
In-house 299	1406.0	P
In-house 300	1877.8	P
In-house 301	2074.6	P
In-house 302	1396.1	P
In-house 303	3866.0	P
In-house 304	858.5	N
In-house 305	4715.7	P
In-house 306	15638.8	P
In-house 307	5225.1	P
In-house 308	5346.0	P
In-house 309	1471.2	P
In-house 310	13337.0	P
In-house 311	798.3	N
In-house 312	14559.9	P
In-house 313	6031.5	P
In-house 314	5673.8	P
In-house 315	22860.4	P
In-house 316	4634.2	P
In-house 317	19099.9	P
In-house 318	3672.7	P
In-house 319	19637.6	P
In-house 320	4549.0	P

Compound Name	RI integrated area (count)	Positive (P) / Negative (N)
In-house 321	1129.6	P
In-house 322	5896.7	P
In-house 323	5941.6	P
In-house 324	17099.3	P
In-house 325	15824.5	P
In-house 326	4531.9	P
In-house 327	15509.1	P
In-house 328	2694.4	P
In-house 329	901.7	N
In-house 330	3031.2	P
In-house 331	12404.6	P
In-house 332	1496.9	P
In-house 333	3865.7	P
In-house 334	1266.1	P
In-house 335	1538.9	P
In-house 336	2784.2	P
In-house 337	2810.2	P
In-house 338	6882.1	P
In-house 339	6897.0	P
In-house 340	4545.2	P
In-house 341	13212.3	P
In-house 342	6363.1	P
In-house 343	12436.4	P
In-house 344	2636.0	P
In-house 345	2246.6	P
In-house 346	806.7	N
In-house 347	2421.0	P
In-house 348	2068.7	P
In-house 349	2266.7	P
In-house 350	1346.7	P

Compound Name	RI integrated area (count)	Positive (P) / Negative (N)
In-house 351	220.2	N
In-house 352	19322.0	P
In-house 353	5957.0	P
In-house 354	1419.8	P
In-house 355	20522.2	P
In-house 356	6501.4	P
In-house 357	5952.7	P
In-house 358	11560.2	P
In-house 359	3532.3	P
In-house 360	14321.3	P
In-house 361	1956.9	P
In-house 362	5563.4	P
In-house 363	1439.6	P
In-house 364	6793.5	P
In-house 365	2543.4	P
In-house 366	2151.7	P
In-house 367	2375.6	P
In-house 368	1678.7	P
In-house 369	1513.8	P
In-house 370	3645.4	P
In-house 371	1320.1	P
In-house 372	3380.3	P
In-house 373	523.6	N
In-house 374	2014.4	P
In-house 375	972.8	N
In-house 376	3435.5	P
In-house 377	1783.0	P
In-house 378	824.6	N
In-house 379	1915.6	P
In-house 380	1806.7	P

Compound Name	RI integrated area (count)	Positive (P) / Negative (N)
In-house 381	2795.7	P
In-house 382	3882.0	P
In-house 383	4097.5	P
In-house 384	1906.9	P
In-house 385	1940.6	P
In-house 386	7993.1	P
In-house 387	1898.4	P
In-house 388	2130.9	P
In-house 389	711.6	N
In-house 390	1496.3	P
In-house 391	8257.7	P
In-house 392	824.9	N
In-house 393	937.4	N
In-house 394	10302.6	P
In-house 395	3975.9	P
In-house 396	33670.4	P
In-house 397	2246.1	P
In-house 398	318.8	N
In-house 399	403.2	N
In-house 400	114.0	N
In-house 401	345.8	N
In-house 402	358.4	N
In-house 403	805.5	N
In-house 404	622.5	N
In-house 405	311.5	N
In-house 406	137.2	N
In-house 407	59.0	N
In-house 408	16879.5	P
In-house 409	304.2	N
In-house 410	672.6	N

Compound Name	RI integrated area (count)	Positive (P) / Negative (N)
In-house 411	581.7	N
In-house 412	1503.2	P
In-house 413	2363.5	P
In-house 414	3016.0	P
In-house 415	564.1	N
In-house 416	1478.8	P
In-house 417	1020.2	P
In-house 418	149.9	N
In-house 419	456.6	N
In-house 420	100482.3	P
In-house 421	1027.2	P
In-house 422	1555.9	P
In-house 423	2004.9	P
In-house 424	18548.5	P
In-house 425	910.0	N
In-house 426	3603.9	P
In-house 427	5867.3	P
In-house 428	3730.5	P
In-house 429	567.9	N
In-house 430	481.5	N
In-house 431	770.4	N
In-house 432	164.6	N
In-house 433	627.9	N
In-house 434	529.6	N
In-house 435	473.1	N
In-house 436	3842.0	P

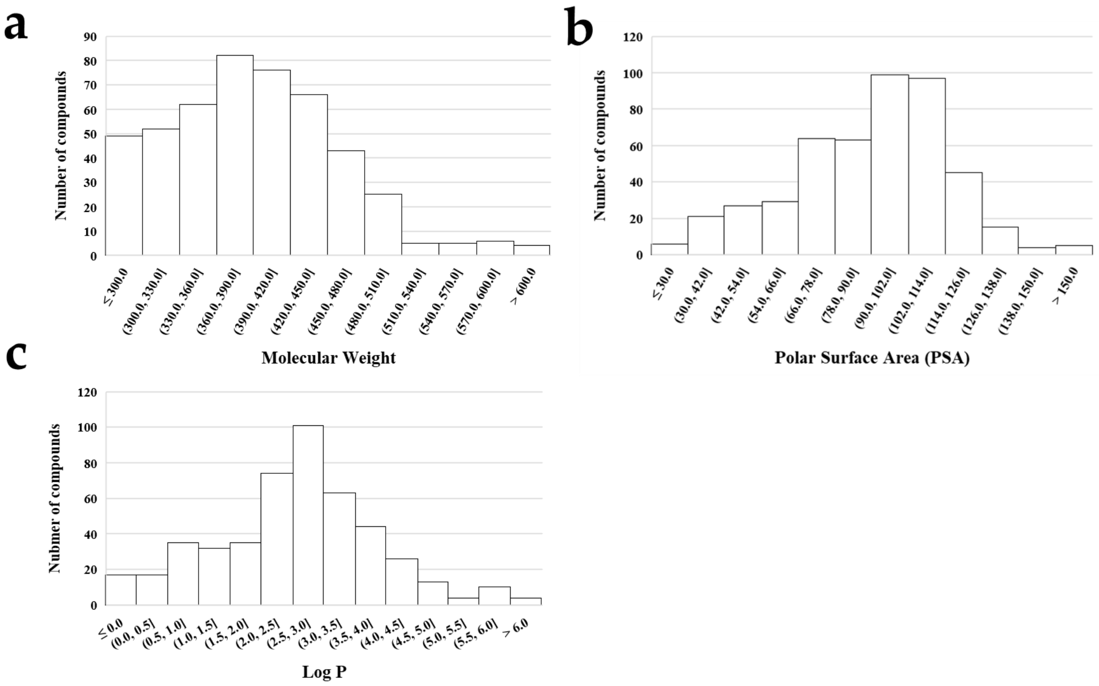

Figure S1. Distribution of Physicochemical Parameters

Molecular Weight (A), Polar Surface Area (PSA) (B), Log P (C) of 475 compounds used for QSAR modeling.

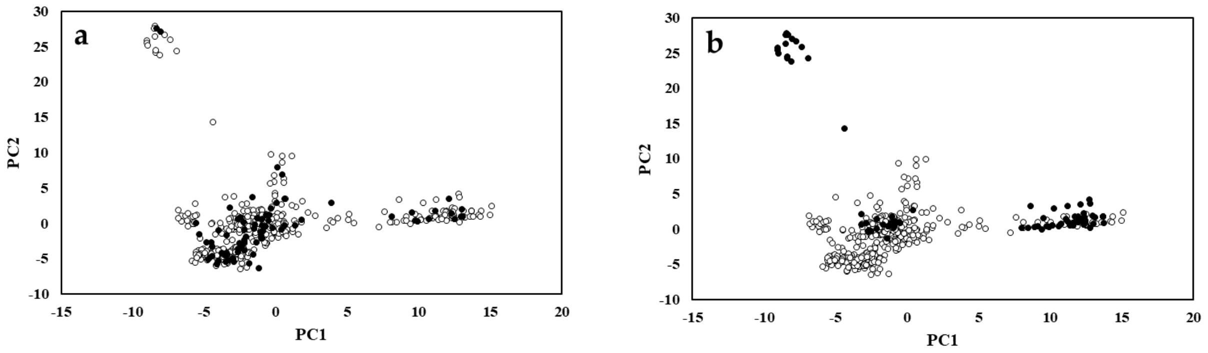

Figure S2. PCA plot of training and test dataset using Extended Connectivity FingerPrint 4 (a: Random-split b: Time-split)

PCA was conducted using PCA function from scikit-learn (ver. 1.2.2) library in python (ver. 3.11.8). In python, the compound structure in canonical SMILES format was read, Extended Connectivity FingerPrint 4 (ECFP4) was calculated as a fingerprint, and after normalization PCA analysis was performed with two components. White and black circles indicate compounds used in the training and external datasets, respectively. The contribution ratio of PC1 and PC2 is 3.4%.
